# Uptake of family planning methods and unplanned pregnancies among HIV-infected individuals: a cross-sectional survey among clients at HIV clinics in Uganda

**DOI:** 10.1186/1758-2652-14-35

**Published:** 2011-06-30

**Authors:** Rhoda K Wanyenze, Nazarius M Tumwesigye, Rosemary Kindyomunda, Jolly Beyeza-Kashesya, Lynn Atuyambe, Apolo Kansiime, Stella Neema, Francis Ssali, Zainab Akol, Florence Mirembe

**Affiliations:** 1 Makerere University School of Public Health, Kampala, Uganda; 2 UNFPA, Kampala, Uganda; 3 Makerere University School of Medicine, Kampala, Uganda; 4 Ministry of Health/AIDS Control Program, Kampala, Uganda; 5 Makerere University School of Social Sciences, Kampala, Uganda; 6 Joint Clinical Research Centre, Kampala, Uganda

## Abstract

**Background:**

Prevention of unplanned pregnancies among HIV-infected individuals is critical to the prevention of mother to child HIV transmission (PMTCT), but its potential has not been fully utilized by PMTCT programmes. The uptake of family planning methods among women in Uganda is low, with current use of family planning methods estimated at 24%, but available data has not been disaggregated by HIV status. The aim of this study was to assess the utilization of family planning and unintended pregnancies among HIV-infected people in Uganda.

**Methods:**

We conducted exit interviews with 1100 HIV-infected individuals, including 441 men and 659 women, from 12 HIV clinics in three districts in Uganda to assess the uptake of family planning services, and unplanned pregnancies, among HIV-infected people. We conducted multivariate analysis for predictors of current use of family planning among women who were married or in consensual union and were not pregnant at the time of the interview.

**Results:**

One-third (33%, 216) of the women reported being pregnant since their HIV diagnoses and 28% (123) of the men reported their partner being pregnant since their HIV diagnoses. Of these, 43% (105) said these pregnancies were not planned: 53% (80) among women compared with 26% (25) among men. Most respondents (58%; 640) reported that they were currently using family planning methods. Among women who were married or in consensual union and not pregnant, 80% (242) were currently using any family planning method and 68% were currently using modern family planning methods (excluding withdrawal, lactational amenorrhoea and rhythm). At multivariate analysis, women who did not discuss the number of children they wanted with their partners and those who did not disclose their HIV status to sexual partners were less likely to use modern family planning methods (adjusted OR 0.40, range 0.20-0.81, and 0.30, range 0.10-0.85, respectively).

**Conclusions:**

The uptake of family planning among HIV-infected individuals is fairly high. However, there are a large number of unplanned pregnancies. These findings highlight the need for strengthening of family planning services for HIV-infected people.

## Background

In 2008, an estimated 1.4 million pregnant women in low- and middle-income countries were living with HIV; 90% of these women were from 20 countries, 19 of which were in sub-Saharan Africa [1]. In the same year, 430,000 children were newly infected with HIV, and more than 90% of infections were through mother to child transmission (MTCT). Access to antiretrovirals (ARVs) for prevention of mother to child transmission of HIV (PMTCT) has steadily improved, but remains low, with 45% of HIV-infected, pregnant women in low- and middle-income countries having received antiretroviral drugs to prevent HIV transmission to their children in 2008 [1]. Preventing unintended pregnancies among women living with HIV is the second pillar for PMTCT, but its potential has not been fully utilized [1-3]. In order to address this gap, the World Health Organization (WHO) strategy to accelerate the scale up of HIV prevention, care and treatment for women and children includes promotion and support for the integration of HIV prevention, care and treatment services within maternal, newborn and child health and reproductive health programmes [1].

Uganda is one of the top 10 countries in terms of having the highest numbers of HIV-infected pregnant women [1]. It is estimated that about 110,000 new HIV infections occurred in Uganda in 2008, approximately one-fifth as a result of MTCT [4]. Studies have documented the effectiveness of family planning (FP) in preventing vertical transmission of HIV [5-7]. However, FP uptake and utilization in Uganda has remained low. The Uganda Demographic Health Survey in 2006 estimated that only 42% of women had ever used contraceptives and 24% were currently using contraceptives [8]. The unmet need for FP among women in Uganda remains high, estimated at 41% of women in reproductive age groups. The total fertility rate has also remained high and stagnant over the past decade, and is currently estimated at 6.7 [8,9].

Delivery of FP services for HIV-infected individuals in Uganda is still inadequate largely due to the parallel nature of FP and HIV services [10]. National plans and guidelines encourage the integration of sexual and reproductive health (SRH), including FP with HIV services, as a key intervention to reduce HIV transmission [9,11,12]. However, most PMTCT interventions have largely focused on the provision of ARVs for prophylaxis with limited attention to prevention of unintended pregnancies. The uptake of FP among HIV-infected individuals, and their preferences and hindrances in uptake and utilization of FP services, was not fully understood. The aim of this study was to assess the utilization of FP services and unintended pregnancies among HIV-infected men and women in Uganda. The study was conducted as part of a larger study that was intended to inform the integration of sexual and reproductive health and HIV services.

## Methods

### Study sites

The study was conducted in 12 HIV clinics in the districts of Gulu, Kabarole and Kampala, including both urban and rural sites. The clinics included a HIV clinic within the national referral hospital in Mulago (Mulago HIV clinic), two public regional referral hospitals (Gulu and Fort Portal), five level IV health centres (Kiswa, Bukuku, Kibito, Lalogi and Awach) and four non-public facilities, including the Joint Clinical Research Centre (Kampala branch), The AIDS Support Organisation (Gulu branch), Nsambya Home Care, and Virika Hospital.

The selection of sites was intended to capture the lower-level facilities (health centres) and the higher-level facilities (hospitals), as well as non-public facilities providing HIV care. The healthcare delivery system in Uganda is hierarchically organized from health centre (HC) II to HC IV and district hospitals. Above the district hospitals are the regional referral and national referral hospitals. The study focused on HIV clinics and not family planning facilities because the primary aim of the larger study was to assess the integration of SRH services into HIV clinics. In terms of the geographical spread, the selection aimed to include the districts in northern Uganda that experienced insecurity with disruption of service delivery for several years, and the more stable southern districts, as well as the urban areas (capital city of Kampala).

### Study design and data collection procedures

The study was cross-sectional by design and the data was collected using interviewer-administered, face-to-face interviews. Interviews were conducted in English or the local languages, including Luganda, Luo and Rutoro, depending on the preferences of the respondents. The questionnaires were pilot tested in all languages prior to data collection. The participants were people living with HIV (PLHIV) attending the selected facilities on the day of the interview. Data analysis included 1100 respondents. The inclusion criteria for this analysis were: (1) age (women and men within the age bracket of 15-49 years); (2) clients who had attended the health facility for at least six months; and (3) patients who were not too ill (physically and mentally) to provide informed consent and participate in the interviews (based on the judgement of the clinic nurses and interviewers).

The data for this paper was derived from a study whose objectives explored a larger scope of reproductive health issues, including family planning, antenatal clinic attendance and delivery, and cervical cancer screening. All HIV-infected individuals within the reproductive age group (including adolescents) were eligible irrespective of whether or not they were sexually active. Because the larger study from which these data was derived also evaluated client satisfaction with SRH services at the facilities, only clients who had attended the facilities for at least six months were included. In order to avoid double counting, couples were not enrolled in the study.

For the small rural clinics, one of every two patients was systematically sampled, while in the larger urban clinics, one of every four patients was selected. At the Joint Clinical Research Centre and Nsambya Home Care, which had a large number of adolescents in care, one of every four adolescents was selected. For the remaining facilities, all adolescents who attended the HIV clinics during the interview period (July to October 2009) were approached for participation. After sampling, interviewers explained the purpose of the study and conducted eligibility screening. Eligible individuals (including the adolescents) who agreed to participate provided written consent (signature or thumb print).

Whereas adolescents who acquired HIV infection through MTCT would find it easier to involve their parents, those who acquired HIV sexually and whose parents may not have been aware that they were HIV infected would find it difficult to do so. Similarly, adolescents who were not living with their biological parents would find it difficult to involve their guardians if they had not disclosed their HIV status to the guardians. Because of these considerations, adolescents provided consent, but were given the option of involving their parents and/or guardians in the consent process. The study was approved by the Mengo Hospital Ethics Committee and the National Council for Science and Technology.

### Measures

In addition to the socio-demographic characteristics, interviews elicited information on the number of pregnancies (current and previous, and pregnancies since the respondents were diagnosed with HIV). Other variables included: knowledge and use of FP; preferred contraceptive options for future use (for both respondents who were using and those who were not using FP); number of live biological children; fertility desires and intentions of the respondents and their sexual partners; discussion of the number of children, as well as timing of pregnancy with sexual partners; and disclosure of HIV status to their sexual partners. For fertility desires, respondents were asked to grade their and their partner's desires for children into none, low, medium or high. We also collected information on the health status of the respondents, whether they were on antiretroviral therapy and duration on treatment, and duration of time since HIV diagnosis. For health status, respondents were asked to rate their health status as poor, fair, good or very good.

### Data analysis

We conducted univariate and bivariate analysis to determine the proportion of men and women who reported current use of FP methods by gender. We also calculated the proportion of men and women who used dual methods (condoms and other methods), as well as those who used other methods without condoms. We conducted analysis for unplanned pregnancies and fertility desires among men and women. Additionally, we calculated the proportion of women who were married or in consensual union and not pregnant, and who were currently using FP methods, by socio-demographic and other characteristics. The women in union included all women who were sexually active (reported sexual contact within 12 months of the interview). In the general description of FP use, we included FP methods that the men reported that their sexual partners were using. However, in the bivariate and multivariate analysis for predictors of current FP use, only women who were married or in consensual relationship and were not pregnant at the time of the interview were included since such women were potentially at risk of becoming pregnant.

We calculated the proportion of women who were currently using any FP method, the proportion who were currently using modern methods (excluding lactational amenorrhoea, rhythm, and withdrawal), and the proportion who were using effective FP methods (modern methods, excluding condoms). The outcome variable for the bivariate and multivariate analysis was current use of modern FP methods (including condoms).

All background characteristics of the respondents were tested for significance of relationship with current use of modern FP methods. Variables that were significant or with borderline significance (p ≤ 0.1) were included into the multivariate model. The variables that were included in the model were eliminated again if they were not found consistently significant in further multivariate analysis. Then a few of the variables that were not significant in the bivariate analysis were included in the model to check whether they added any value in terms of goodness of fit. If they did not add any value, they were eliminated again. Some variables, such as age, were left in the model due to logical importance [13]. Data analysis was done using STATA version 10.

## Results

Of the 1178 individuals who were screened, 1152 (98%) were eligible and of these, 1142 (99%) agreed to participate. Overall, 485 (44%) of the respondents were from the urban and peri-urban areas, (659; 60%) were women, and most were married (505; 46%) or in consensual union (140; 13%). In total, 506 respondents (46%) were within the 30-39 year age group. Adolescents (15-19 years) constituted 69 (6%) of the respondents; 20 of the 69 adolescents had ever had sexual contact. Respondents had various low-paying jobs, such as casual labour and small business, but peasant farming was the most common job (385; 35%). The majority of the respondents (679; 62%), reported earning less than 100,000 Uganda shillings (less than US$50) a month (Table 1). Approximately 70% (772) were taking ARVs and 626 (80%) rated their health status as good or very good.

**Table 1:** Socio-demographic characteristics of the study respondents

Characteristics	Men	(n = 441)	Women	(n = 659)	All	(n = 1100)	P value
	**Freq**	**%**	**Freq**	**%**	**Freq**	**%**	

**Age group**							

15-19	27	6.2	42	6.4	69	6.3	<0.001

20-24	23	5.2	67	10.2	90	8.2	

25-29	48	10.9	141	21.4	189	17.2	

30-34	99	22.5	164	24.9	263	23.9	

35-39	109	24.7	134	20.3	243	22.1	

40-44	79	17.9	89	13.5	168	15.3	

45-49	56	12.7	22	3.3	78	7.1	

**Education level**							

None	46	10.5	130	19.7	176	16.0	<0.001

Primary	237	54.0	348	52.8	585	53.3	

Secondary+	156	35.5	181	27.5	337	30.7	

**District**							

Kampala	135	30.6	239	36.3	374	34.0	

Kabarole	138	31.3	222	33.7	360	32.7	0.02

Gulu	168	38.1	198	30.1	366	33.3	

**Residence**							

Urban	169	38.3	316	48.0	485	44.1	0.002

Rural	272	61.7	343	52.0	615	55.9	

**Marital status**							

Single	46	10.4	70	10.6	116	10.6	<0.001

In relationship	40	9.1	100	15.2	140	12.7	

Married	261	59.2	244	37.0	505	45.9	

Divorced/separated	60	13.6	111	16.8	171	15.6	

Widowed	34	7.7	134	20.3	168	15.3	

**Religion**							

Catholic	228	51.7	324	49.2	552	50.2	0.155

Protestant	131	29.7	187	28.4	318	28.9	

Muslim	36	8.2	48	7.3	84	7.6	

Other	46	10.4	100	15.1	146	13.3	

**Occupation**							

Peasant farmer	143	32.4	240	36.4	383	34.8	0.001

Salaried	60	13.6	55	8.4	115	10.5	

Business/commercial	95	21.5	162	24.6	257	23.4	

Casual worker	71	16.1	61	9.3	132	12.0	

Other	72	16.1	141	21.4	212 5	19.4	

**Expenditure**							

<30,000	118	26.8	215	32.6	333	30.3	0.024

31,000-100,000	136	30.8	210	31.9	346	31.5	

110,000+	160	36.3	185	28.1	345	31.4	

Don't know/missing/can't disclose	27	6.2	49	7.4	76	6.9	

**Health facility**							

Hospital	243	55.1	363	55.1	606	55.1	0.922

HC IV	108	24.5	156	23.7	264	24.0	

Other	90	20.4	140	21.2	230	20.9	

**Number of years since testing HIV+ve**							

<1	64	14.7	61	9.3	125	11.4	0.02

1-2	198	45.3	283	43.1	481	44.0	

3-4	113	25.9	198	30.1	311	28.4	

5+	62	14.2	115	17.5	177	16.2	

Time since started on ARVs (yrs)*							

<1	81	24.9	84	18.8	165	21.4	0.115

1-4	197	60.6	297	66.4	494	64.0	

5+	47	14.5	66	14.8	113	14.6	

**Health status**							

Poor/fair	70	21.3	84	18.6	154	19.7	

Good	180	54.9	251	55.5	431	55.3	

Very good	78	23.8	117	25.9	195	25.0	0.58

**Number of children**							

0-2	115	32.1	166	34.5	281	33.4	

3-4	117	32.7	187	38.7	304	36.2	0.03

5-15	126	35.2	130	26.9	256	30.4	

**Disclosure of HIV status**							

Yes	289	65.5	317	48.1	606	55.1	

No	25	5.7	54	8.2	79	7.2	<0.001

No partner	127	28.8	288	43.7	415	37.7	

**Been pregnant since HIV diagnosis**							

Yes	123	27.9	216	32.8	339	30.8	

No	318	72.1	443	67.2	761	69.2	0.09

Pregnant/partner pregnant**							

Yes	33	11.3	44	13.1	77	12.2	

No	260	88.7	292	86.9	552	87.8	0.48

**Desire to have children rated as medium or high**							

Yes	93	24.4	112	19.0	205	21.1	

No	288	75.6	477	81.0	765	78.9	0.04

*Only 772 were receiving ARVs

** Answered by respondents who had ever had children

### Fertility desires and intentions, and unplanned pregnancies

Overall, 31% (339) reported that they or their partner had been pregnant since they were diagnosed with HIV; 33% of the women had been pregnant and 28% of the men reported that their partners had been pregnant since the men were diagnosed with HIV (Table 1). Of these, 43% (105) said they did not plan the current or last pregnancy: 53% (80) among women compared with 26% (25) among men. Among 629 respondents who had ever had children in their lifetime, 12% (77) were either pregnant or their partner was pregnant at the time of the interview (Table 1). Overall, 20% (180) of the respondents who already had children desired having more children (Table 1). A slightly larger proportion of men (23%; 85) than women (19%; 95) desired more children. Half of the women (182) and 34% (100) of the men said their partners desired having more children.

### Use of family planning methods and contraceptive preferences for future use

Knowledge of FP methods was very high, with more than 98% of men and women having heard of methods used to prevent conception. The majority (87%; 958) had ever used FP and 58% (640) were currently using an FP method. The most commonly used FP method was male condoms (48%; 530): 62% of the men and 39% of the women were using male condoms (Table 2). Overall, 11% (125) of the respondents reported using dual methods (condoms and other FP methods); 12% of the men (54) and 11% of the women (71) used dual methods. On the other hand, 10% (112) used only other FP methods without condoms; 6% (28) of men and 13% (84) of women used other methods without condoms.

**Table 2:** Currently used and preferred contraceptive methods for future use among HIV-infected men and women

Method Method	Currently used any FP methods freq (%)			Preferred FP method for future use freq (%) (%ge)(%ge)		
	**Men **(n **= **441)	**Women **(n = 659)	**All **(n = 1100)	**Men **(n **= **441)	**Women **(n **= **659)	**All **(n = 1100)*

**Male methods**						

Male condoms	272 (61.8)	258 (39.2)	530 (48.2)	355 (80.5)	419 (63.6)	774(70.4)

Male sterilization	0 (0)	0 (0)	0 (0)	65 (14.7)	85 (12.9)	150 (13.6)

**Female methods**						

Injectable	21 (4.8)	58 (8.8)	79 (7.1)	111 (25.2)	228 (34.6)	339 (30.8)

Pill	9 (2.0)	23 (3.5)	32 (2.9)	92 (20.9)	126 (19.1)	218 (19.8)

Implants	11 (2.5)	15 (2.3)	26 (2.4)	81 (18.4)	118 (17.9)	199 (18.1)

Female sterilization	4 (0.9)	9 (1.4)	13 (1.2)	62 (11.1)	114 (17.3)	176 (16.0)

Female condoms	5 (1.1)	5 (0.8)	10 (0.9)	96 (21.8)	129 (19.6)	225 (20.5)

Emergency contraception	3 (0.7)	1 (0.2)	4 (0.4)	65 (14.7)	115 (17.5)	180 (16.4)

IUD	0.0 (0)	1 (0.2)	1 (0.1)	39 (8.8)	45 (6.8)	84 (7.6)

Diaphragm	0 (0)	1 (0.2)	0 (0.09)	33 (7.5)	39 (5.9)	72 (6.6)

Foam/jelly	0 (0.0)	3 (0.5)	3 (0.3)	46 (10.4)	94 (14.3)	140 (12.7)

**Natural methods**						

Withdrawal	23 (5.2)	17 (2.6)	40 (3.6)	102 (23.1)	156 (23.7)	258 (23.5)

Rhythm method	12 (2.7)	18 (2.7)	30 (2.7)	106 (24.0)	136 (20.6)	242 (22.0)

Lactational Amenorrhoea	5 (1.1)	16 (2.4)	21 (1.9)	80 (18.4)	125 (19.0)	205 (18.6)

**Using any FP method**	**300 (68.0)**	**340 (51.6)**	**640 (58.2)**	-	-	-

**Using dual FP methods**	**54 (12.2)**	**71 (10.8)**	**125 (11.4)**			

*Some respondents selected more than one preferred FP method

In terms of preferred FP methods for future use, the majority (70%; 774) still preferred the male condom: 81% of the men and 64% of the women preferred male condoms (Table 2). Other preferences included injectables (31%), female condoms (21%) and implants (18%). Preference for male and female sterilization was also fairly high at 14% and 16%, respectively (Table 2). Many respondents preferred natural methods, including withdrawal (24%), rhythm (22%) and lactational amenorrhea (19%).

Among the women who were married or in consensual union and not pregnant, 80% (242) were currently using any FP method, while 68% (207) were currently using modern FP methods (Table 3). When the condoms are excluded, the proportion of women using effective FP methods drops to 15% (44). Current use of modern FP methods was highest among the women with secondary level education or higher (78%: 67) and the salaried women (83%: 24). Current use of modern methods was lowest among women in Gulu District (58%; 57), Catholics (64%) and those who did not discuss the number and timing of children with their sexual partners (58%; 40). Among the women who did not desire having more children, 70% (110) were currently using modern FP methods, not different from those whose desire for children was rated as medium-high (71%; 49).

**Table 3:** Current use of FP among HIV-infected women (married or in consensual union and not pregnant)

Variable	Currently using any FP methods n = 242 (79.6%)	Currently using modern FP methods† n = 207 (68.1%)	Currently using effective FP methods n = 44 (14.5%)
	**Number (%**	**Number (%)**	**Number (%)**

**Age**			

15-24	37 (78.7)	30 (63.8)	12 (25.5)

25-34	119 (80.4)	101 (68.2)	16 (10.8)

35-49	86(78.9)	76 (69.7)	16 (14.7)

**Education**			

None	42 (73.7)	36 (63.2)	11 (19.3)

Primary	123 (76.4)	104 (64.6)	22 (13.7)

Secondary+	77 (89.5)	67 (77.9)	11 (12.8)

**District**			

Kampala	98 (83.8)	84 (71.8)	13 (11.1)

Gulu	69 (69.7)	57 (57.8)	21 (21.2)

Kabarole	75 (85.2)	66 (75.0)	10 (11.4)

**Religion**			

Catholic	115 (77.2)	95 (63.8)	23 (15.4)

Protestant	71 (82.6)	60 (69.8)	12 (14.0)

Muslim	26 (92.9)	24 (85.7)	3 (10.7)

Other	28 (71.8)	26 (66.7)	6 (15.4)

**Marital status**			

In relationship	78 (83.9)	63 (67.7)	13 (14.0)

Married	164 (77.7)	144 (68.3)	31 (14.7)

**Occupation**			

Peasant farmer	90 (77.6)	76 (65.5)	21 (18.1)

Salaried	27 (93.1)	24 (82.8)	3 (10.3)

Business/commercial	63 (80.8)	52 (66.7)	10 (12.8)

Casual worker	20 (74.1)	18 (66.7)	3 (11.1)

Other	42 (77.8)	37 (68.5)	7 (13.0)

**Expenditure**			

<30,000	62 (72.9)	53 (62.4)	13 (15.3)

31,000-100,000	85 (81.0)	72 (68.6)	15 (14.3)

110,000+	84 (83.2)	71 (70.3)	14 (13.9)

Unknown	11 (84.6)	11 (84.6)	2 (15.4)

**Facility level**			

Hospital	130 (80.3)	112 (69.1)	22 (13.6)

HC IV	61 (80.3)	53 (69.7)	7 (9.2)

Other	51 (77.3)	42 (63.6)	15 (22.7)

**Number of years since testing**			

<1 year	22 (73.3)	19 (63.3)	2 (6.7)

1-2	104 (77.0)	88 (65.2)	24 (17.8)

3-4	65 (83.3)	56 (71.8)	12 (15.4)

5+	50 (83.3)	43 (71.7)	5 (8.3)

**Time since start on ARVs+**			

<1 year	28 (77.8)	26 (72.2)	2 (5.6)

1-4	113 (84.3)	100 (74.6)	15 (11.2)

5+	19 (76.0)	14 (56.0)	2 (8.0)

**Health status**			

Poor/fair	28 (75.7)	24 (64.9)	4 (10.8)

Good	84 (80.0)	76 (72.4)	12 (11.4)

Very good	49 (90.7)	41 (75.9)	4 (7.4)

**Number of biological children**			

None	19 (67.9)	18 (64.3)	3 (10.7)

1-2	92 (84.4)	79 (72.5)	16 (14.7)

3-4	89 (81.7)	74 (67.9)	17 (15.6)

5-10	42 (72.4)	36 (62.1)	8 (13.8)

**Disclosed HIV status**			

Yes	210 (80.2)	180 (68.7)	41 (15.7)

No	32 (19.8)	25 (62.5)	3 (7.5)

**Desire for children**			

None	132 (83.5)	110 (69.6)	23 (14.6)

Low	54 (73.0)	45 (60.8)	13 (17.6)

Medium/high	53 (76.8)	49 (71.0)	8 (11.6)

**Partner desire for children**			

None	77 (87.5)	67 (76.1)	5 (5.7)

Low	33 (68.8)	29 (60.4)	8 (16.7)

Medium/high	132 (78.6)	111 (66.1)	31 (18.5)

**Discussion on timing of children**			

Yes	190 (83.3)	163 (71.5)	32 (14.0)

No	48 (68.6)	40 (57.1)	11 (15.7)

**† **Effective methods (excludes condoms, withdrawal, lactational amennorroea and rhythm)

### Predictors of current use of modern family planning methods

At bivariate analysis, women from Gulu were less likely to use modern FP methods than those from Kampala District (OR 0.53, 0.30-0.94), Moslem women were more likely to use FP than Catholic women (OR 3.41, 1.12-10.35), and women who did not discuss the number and timing of children with sexual partners were less likely to use FP (OR 0.53, 0.31-0.93). There was no significant association between desire to have children by respondent or sexual partner, number of live children and current use of FP (Table 4). Similarly, there was no significant association between health status, being on ARVs, duration on treatment, and current use of FP (Table 4).

**Table 4:** Predictors of current use of modern FP methods among HIV-infected women (married or in consensual union and not pregnant)

	Unadjusted OR	95%CI	Adjusted OR	95%CI
**Age (base = 15-24)**				

25-34	1.21	0.61-2.42	0.18	0.03-0.97

35-49	1.31	0.63-2.69	0.21	0.04-1.11

**Education (base = none)**				

Primary	1.06	0.57-1.99		

Secondary+	2.06	0.98-4.32		

**District (base = Kampala)**				

Gulu	0.53	0.30-0.94*	0.42	0.15-1.15

Kabarole	1.18	0.63-2.21	0.97	0.42-2.21

**Religion (base = Catholic)**				

Protestant	1.31	0.74-2.32	1.48	0.68-3.21

Muslim	3.41	1.12-10.35*	3.18	0.77-13.10

Other	1.14	0.54-2.39	1.49	0.51-4.36

**Marital status (base = in relationship)**				

Married	1.02	0.61-1.73		

**Occupation (base = peasant)**				

Salaried	2.52	0.90-7.12		

Business/commercial	1.05	0.57-1.93		

Casual worker	1.05	0.43-2.56		

Other	1.15	0.57-2.28		

**Expenditure (base <30,000)**				

31,000-100,000	1.32	0.72-2.40		

110,000+	1.43	0.77-2.64		

**Facility level (base = hospital)**				

HC IV	1.03	0.57-1.86		

Other	0.78	0.43-1.43		

**Number of years since testing (base < 1)**				

1-2	1.08	0.48-2.47		

3-4	1.47	0.60-3.59		

5+	1.46	0.58-3.72		

**Time since start on ARVs+ (base <1)**				

1-4	1.13	0.49-2.59	1.32	0.54-3.24

5+	0.49	0.17-1.43	0.45	0.13-1.53

**Health status (base = poor/fair)**				

Good	1.42	0.64-3.16		

Very good	1.71	0.68-4.28		

**Number of children (base = 0)**				

1-2	1.46	0.61-3.52		

3-4	1.17	0.49-2.81		

5-10	0.91	0.36-2.32		

**Disclosed HIV status (base = yes)**				

No	0.76	0.38-1.52	0.30	0.10-0.85*

**Desire for children (base = none)**				

Low	0.68	0.38-1.21		

Medium/high	1.07	0.57-1.99		

**Partner desire for children (base = none)**				

Low	0.48	0.22-1.02	0.34	0.12-0.97*

Medium/high	0.61	0.34-1.10	0.45	0.20-1.03

**Discussion on timing of children (base = yes)**				

No	0.53	0.31-0.93*	0.40	0.20-0.81*

* p < = 0.05

At multivariate analysis, women who classified their partners' desire for children as low were less likely to use modern FP compared with those who classified partners' desire as high (adjusted OR 0.34, 0.12-0.97). Women who had not disclosed their HIV status to their sexual partners and those who did not discuss the number and timing of children with their sexual partners were also less likely to use modern FP methods (Adj OR 0.30, 0.10-0.85, and 0.40, 0.20-0.81, respectively (Table 4).

## Discussion

These findings show that current use of family planning methods among HIV-infected individuals in care was higher than that reported in the general population in Uganda, which is estimated at 24% [8]. The higher uptake of FP among this population was due to high condom use rates [12]. When condoms were excluded, the current use of modern methods among women dropped from 68% to 15%. Condom use is encouraged by providers for prevention of sexual transmission among HIV-infected individuals. Many HIV-infected individuals may thus opt for condom use for sexual prevention of HIV, as well as FP.

Despite the high rates of current use of FP methods, the proportion reporting unintended pregnancies was high. The large number of unintended pregnancies among HIV-infected individuals in Uganda and elsewhere has also been reported by other studies [5,14,15]. In this population, use of less effective FP methods, including condoms, withdrawal, lactational amenorrhea and rhythm, may be partly responsible for the large number of unintended pregnancies. Use of the most effective methods, including implants and female and male sterilization, was very low.

Incorrect and/or inconsistent use of FP methods also reduces the effectiveness of the user-dependent contraceptive methods. Male condoms provide dual protection from HIV transmission and acquisition, as well as pregnancy. However, the effectiveness reduces when the condoms are not used correctly and consistently [16-18]. Also, the effectiveness of some hormonal contraceptives may be reduced due to interaction with antiretroviral drugs [17]. However, the non-use of contraceptives remains a bigger contributor to unintended pregnancies in comparison to failure of contraceptive methods [18]. The proportion of women reporting unplanned pregnancies was twice that among men. Because of the much higher condom use rates among men, overall FP use for men (68%) was higher than that among women (52%).

Dual protection, which refers to the simultaneous protection against HIV and other sexually transmitted infections and pregnancy, can be achieved by correct use of condoms with or without other effective methods of contraception. Use of condoms simultaneously with other effective contraceptive methods among HIV-infected individuals increases protection against pregnancy, in addition to prevention of HIV transmission and acquisition. However, dual use of FP methods was very low in this population. Efforts to reduce unintended pregnancies among HIV-infected individuals should address increased uptake of contraceptives coupled with selection of more effective methods, as well as support to ensure correct and consistent use of the user-dependent methods and dual protection. Increased sensitization and training of providers is also required to improve the quality of FP services.

Preference for the female condom was fairly high at 21%, although female condoms were not available on the Ugandan market at the time of this study. Additionally, about one-third of women preferred injectables and implants, and a significant proportion preferred sterilization. Availing these FP options for PLHIV on site or through linkages with other providers, could increase the uptake and use of FP methods and reduce unplanned pregnancies for more than 70% of men and women who did not desire having more children. Preference for less effective FP methods and methods that do not provide dual protection should be addressed through patient education. Patient education and support should also address increased communication between spouses on the need to avoid unintended pregnancies through FP use, timing and spacing of pregnancies, contraceptive options, as well as disclosure of HIV status to sexual partners.

Discussion of HIV status among couples may be easier when they have disclosed their HIV status. On the other hand, a significant proportion of the respondents (21%) reported that they or their partners wished to have more children, which requires planning and support to ensure that the children are protected. PLHIV have a right to have children if they choose to do so. However, they should be supported to ensure that the children are born free of HIV, in addition to prevention of transmission to sexual partners in cases of sero-discordance.

Several studies show that knowledge of one's HIV status is associated with a desire to limit childbearing with contraceptive use [19-21]. Gaps in the provision of FP services for HIV-infected individuals have remained due to the parallel nature of the two services [22,23]. However, a shift in thinking has recently been achieved, with collaborations established at international levels between the major players [22]. This presents a great opportunity for the scale up of integrated SRH services for HIV-infected individuals if the operational challenges, including strengthened health systems, can be addressed.

### Study limitations

This study was cross-sectional in nature, and did not include questions on why the respondents who did not desire having children were not using contraceptives, and on the number of dead children, and did not distinguish between the level of desire for children. These aspects need further exploration. The study recruited PLHIV who had been in HIV care for at least six months. Most of the sites included in the study are also funded by the US President's Emergency Plan for AIDS Relief (PEPFAR) and have integrated SRH services in line with PEPFAR guidelines, as well as national guidelines. Also, the facilities may have had varying levels of availability of FP supplies. The findings from this study may therefore not be generalized to all PLHIV or all HIV care and treatment sites within Uganda.

However, the study indicates that once diagnosed and enrolled in care, the uptake of FP among HIV-infected individuals can be increased dramatically [8,24]. Ensuring improved SRH services, including FP, in the general population remains a priority since the majority of HIV-infected individuals remain unaware of their HIV status.

## Conclusions

The findings of this study highlight the need for integration and strengthening of FP services for PLHIV. HIV prevention, care and treatment services should incorporate sexual and reproductive health services, including FP as an integral component. The preferred FP methods should be available at HIV service delivery sites or through linkages with other providers.

## Competing interests

The authors declare that they have no competing interests.

## Authors' information

Rhoda Wanyenze is Program Manager at the Makerere University School of Public Health-CDC Fellowship Program. Nazarius Mbona Tumwesigye is a Senior Lecturer at the Makerere University School of Public Health. Rosemary Kindyomunda is National Programme Officer HIV/AIDS, United Nations Population Fund. Jolly Beyeza is a Consultant for the Department of Obstetrics and Gynaecology, Mulago Hospital. Lynn Atuyambe is Lecturer at the Makerere University School of Public Health. Apolo Kansiime works in the AIDS Control Programme, Ministry of Health, Uganda. Stella Neema is a Senior Lecturer in the Department of Sociology, Makerere University. Francis Ssali is Head of Clinical Services, Joint Clinical Research Centre. Zainab Akol is Program Manager of the AIDS Control Programme, Ministry of Health, Uganda. Florence Mirembe is Professor of Obstetrics and Gynaecology, Makerere University.

## Authors' contributions

All authors participated in the design of the study and development of the questionnaires. Additionally, RW, NMT, LA, SN and FM conducted the data analysis. All the authors have read and approved the final manuscript.
